# Preliminary analysis using multi-atlas labeling algorithms for tracing longitudinal change

**DOI:** 10.3389/fnins.2015.00242

**Published:** 2015-07-14

**Authors:** Regina E. Y. Kim, Spencer Lourens, Jeffrey D. Long, Jane S. Paulsen, Hans J. Johnson

**Affiliations:** ^1^Department of Psychiatry, University of IowaIowa City, IA, USA; ^2^Department of Biostatistics, College of Public Health, University of IowaIowa City, IA, USA; ^3^Department of Neurology, Carver College of Medicine, University of IowaIowa City, IA, USA; ^4^Neuroscience, Carver College of Medicine, University of IowaIowa City, IA, USA; ^5^Department of Electrical Engineering, University of Iowa, Iowa CityIA, USA; ^6^Biomedical Engineering, University of IowaIowa City, IA, USA

**Keywords:** brain MRI, longitudinal data analysis, multicenter study, machine learning, multi-atlas label fusion, validation

## Abstract

Multicenter longitudinal neuroimaging has great potential to provide efficient and consistent biomarkers for research of neurodegenerative diseases and aging. In rare disease studies it is of primary importance to have a reliable tool that performs consistently for data from many different collection sites to increase study power. A multi-atlas labeling algorithm is a powerful brain image segmentation approach that is becoming increasingly popular in image processing. The present study examined the performance of multi-atlas labeling tools for subcortical identification using two types of *in-vivo* image database: Traveling Human Phantom (THP) and PREDICT-HD. We compared the accuracy (Dice Similarity Coefficient; DSC and intraclass correlation; ICC), multicenter reliability (Coefficient of Variance; CV), and longitudinal reliability (volume trajectory smoothness and Akaike Information Criterion; AIC) of three automated segmentation approaches: two multi-atlas labeling tools, MABMIS and MALF, and a machine-learning-based tool, BRAINSCut. In general, MALF showed the best performance (higher DSC, ICC, lower CV, AIC, and smoother trajectory) with a couple of exceptions. First, the results of accumben, where BRAINSCut showed higher reliability, were still premature to discuss their reliability levels since their validity is still in doubt (DSC < 0.7, ICC < 0.7). For caudate, BRAINSCut presented slightly better accuracy while MALF showed significantly smoother longitudinal trajectory. We discuss advantages and limitations of these performance variations and conclude that improved segmentation quality can be achieved using multi-atlas labeling methods. While multi-atlas labeling methods are likely to help improve overall segmentation quality, caution has to be taken when one chooses an approach, as our results suggest that segmentation outcome can vary depending on research interest.

## Introduction

Brain MRI analysis from longitudinal multicenter studies has become increasingly important in clinical studies of normal aging as well as in neurodegenerative disorders, such as Huntington (HD), Alzheimer's, and Parkinson's disease. Precise assessment of longitudinal changes of brain structures may provide a non-invasive means to monitor treatment effects of clinical intervention. During the last decade, many large-scale multicenter longitudinal studies have collected series of imaging data (Jack et al., 2008; Paulsen et al., 2008; Tabrizi et al., 2014) to understand how the human brain changes in the course of aging and disease progression. These studies of structural brain changes have provided a key insight into healthy development (Sullivan et al., 2011; Treit et al., 2013; Herting et al., 2014), normal aging (Tang et al., 2001; Resnick et al., 2003; Scahill et al., 2003; Mungas et al., 2005; Risacher et al., 2010), and disease progression (Ahdidan et al., 2011; Tabrizi et al., 2012; Takahashi et al., 2012; Weiner et al., 2012).

We have utilized two independent data sets to compare performance of automated segmentation methods in human MRI. The PREDICT-HD database provides multi-center longitudinal data collected for pre-symptomatic gene-positive HD (Pre-HD) individuals over a 10-year period (Paulsen et al., 2006). Traveling Human Phantom (THP) data was collected for the multicenter reliability study and includes repeated multi-modal MRIs (T1-weighted and T2-weighted) from same five healthy subjects at eight different sites that had either a Siemens 3T TIM Trio scanner or a Philips 3T Achieva scanner (Magnotta et al., 2012). The THP data provides valuable reproducibility insights since the same five individuals traveled to the eight different sites in a month, where no brain change was expected. In this study, PREDICT-HD data was used to compare segmentation accuracy and longitudinal reliability; and THP was used to investigate intra-subject multicenter reliability. We further limited our attention to subcortical structures, which are the main regions of interest in HD. The subcortical structures of interest include the accumben nucleus, caudate nucleus, putamen, globus pallidus, thalamus, and hippocampus. Volume changes in the basal ganglia have been repeatedly reported in several studies in Pre-HD subjects (Paulsen et al., 2014a,b) and HD patients (Rosas et al., 2011; Tabrizi et al., 2013) and are primarily considered part of the hypothesized main brain target region in HD pathology (Phillips et al., 2014).

The interpretation of volumetric change is greatly affected by the quality of the segmentation approach under consideration. It is easily agreeable that large-scale longitudinal data are potentially more powerful but also more prone to methodological bias. Therefore, volume measures for longitudinally collected MRIs are required to accurately capture how individual differences are related to brain structural changes over time. While the large-scale multicenter longitudinal design is increasingly popular, one main factor that limits the sensitivity of multicenter longitudinal studies is data variation. Variation in MRI-driven volumetric measures may result from biological differences between subjects as well as from image characteristic differences, such as intensity profiles, which depend on scanner types, acquisition protocols, field strength, and subject placement in the scanner. The challenge remains in providing segmentation techniques that work in all cases, regardless of type of scanner, progression of disease, or MRI protocol to identify biomarkers which model disease progression and to predict clinical outcomes.

A desire to attain precise and sensitive measurements of MRI-driven volume changes has spurred the creation of several MRI segmentation methods (Balafar et al., 2010). This continual development effort achieved reasonable cross sectional brain anatomy segmentation by adopting a single atlas-based labeling method (Cabezas et al., 2011), which identifies regions of interests by propagating atlas information to a target subject using an image registration technique. This single atlas-based approach requires that the brain morphology presented in the image be very similar between the target subject and the atlas MRI. The single atlas-driven approach becomes vulnerable when inter-image (subject) differences are too large to be captured by any given registration method. The issue becomes increasingly problematic for large-scale data or analysis, where data variation is inherently large while the method solely relies on one atlas.

To overcome the above-mentioned issues of a single atlas-based approach, a multi-atlas labeling approach has been proposed (Rohlfing and Maurer, 2004). The multi-atlas labeling method employs several different atlases to cover a variety of MR data characteristics. Utilization of multiple atlases in a segmentation method takes account for image profile differences and large intersubject anatomical variation that naturally occurs in the human brain (Cabezas et al., 2011). There is also increasing evidence that multi-atlas labeling improves segmentation accuracy in several studies (Wang et al., 2012; Chakravarty et al., 2013; Sjoberg and Ahnesjo, 2013), and consequently, this approach is rapidly gaining popularity (Sabuncu et al., 2010; Zhang et al., 2011a; Jimenez del Toro and Muller, 2014).

The present study was designed to systematically contrast three methods for subcortical segmentation on identical data sets. Three publicly available open-source segmentation tools are utilized in this comparative study: BRAINSCut (Kim et al., 2014), ANTs MALF (Wang et al., 2012), and 3DSlicer's MABMIS (Jia et al., 2012) (Also see Table 1). We are specifically interested in how tools perform on *in-vivo* human MRI studies with respect to at least three aspects: segmentation accuracy, multi-center reliability, and longitudinal reliability. To capitalize on our knowledge and experience in automated segmentation tools, we have evaluated our in-house tool, BRAINSCut, in addition to two distinct techniques that are based on the multi-atlas labeling approach: MABMIS and MALF.

**Table 1:** **A brief summary of three automated segmentation tools investigated in this study: MALF (Wang and Yushkevich, 2013), MABMIS (Jia et al., 2012), and BRAINS Cut (Kim et al., 2014)**.

**Tool**	**General approach**	**Remark**
MALF	Multi-atlas labeling based	Joint fusion algorithm with Advanced SyN-based registration (Avants et al., 2008)
MABMIS	Multi-atlas labeling based	Expedite multiple registrations using a tree-based group-wise registration method and naïve label voting
BRAINS Cut	Machine-learning based	Machine-learning, specifically a Random-forest-based method, which outperformed other classification methods in multicenter large-scale MRI processing in terms of segmentation accuracy and generalizability of large scale data

BRAINSCut is an open-source machine-learning-based segmentation software targeted for processing of multicenter large-scale MRI. The core of the segmentation algorithm implements a machine-learning technique called random-forest to delineate target structures. BRAINSCut excels in processing large-scale multicenter data reliably and efficiently, and has been used extensively by the PREDICT-HD (Paulsen et al., 2013) and TRACK-ON (Tabrizi et al., 2009) research teams. The latest version of BRAINSCut was evaluated using both PREDICT-HD and TRACK-ON data to assess its accuracy and multi-center reliability (Kim et al., 2014).

Multi-atlas based multi-image segmentation (MABMIS) (Jia et al., 2012) proposes an efficient way to expedite multiple registrations between target and atlases. MABMIS aims to address the bottleneck of multi-atlas labeling methods: computationally expensive registrations from multiple atlases to a target image. MABMIS reduces registration time by constructing a hierarchical registration tree between the atlas and target images.

Finally, multi-atlas based label fusion (MALF) provides a great implementation of the multi-atlas labeling approach in conjunction with the advanced normalization tools (ANTs) development framework. The MALF algorithm advances segmentation accuracy via weighted voting, assuming conditional independence between atlases. The approach utilizes ANTs symmetric image normalization (SyN)-based registration (Avants et al., 2008), endowing it with great potential to be a powerful tool in the field. The parameter profiles of MALF are well explained in Wang and Yushkevich (2013) and its performance is formally reported in Yushkevich et al. (2012).

This paper aims to provide validation for several aspects regarding assessment of automated segmentation performance, potentially leading to more powerful tool development in the future. Although we believe that key indicators of the quality of automated segmentation outcomes are their accuracy and reliability, only a few studies address both accuracy and reliability, and their assessment is often limited to short-term period data (Babalola et al., 2009; Wonderlick et al., 2009). Segmentation accuracy warrants the validity of the identified structures to be used for brain research as their definition corresponds to the research intent. On the other hand, reliability means the extent of measurement stability, e.g., across sites (multicenter reliability) or across time (longitudinal reliability), so that outcomes can be used to detect differences between groups or over time. Validity requires that the measurement is reliable, but the measurement can be reliable without being valid (Kimberlin and Winterstein, 2008). Therefore, we sought to investigate both aspects of segmentation quality, accuracy and reliability, in order to compare the three different approaches.

Finally, a sample size analysis was carried out to establish the minimum sample size necessary for detecting changes at the caudate nucleus and putamen level presented in PREDICT-HD MRI data with 80% power. These regions were used because they are established as the most prominent candidates for measuring longitudinal change in HD (Paulsen et al., 2014a). This sample size estimation provides crucial information to give one an idea of what to expect with the current tools available in the field as well as to guide future direction of tool development and study design.

Thus, the goal of this technical report is to provide insight into performance of different brain MRI segmentation approaches, including two emerging multi-atlas labeling techniques, focusing on *in-vivo* longitudinal multicenter MRI data. With the growing demand for a reliable segmentation technique and with attention to multi-atlas labeling methods spreading, this technical report investigated how multi-atlas labeling works on a multicenter longitudinal MRI data set. By utilizing the multicenter longitudinal data, we present quantitative and qualitative assessments of how individual trajectories of subcortical volume relate to the choice of methodology. Although this study utilized the PREDICT-HD data set, the outcomes of this study are generalizable to other study domains involving longitudinal and/or multicenter MRI studies. We hope that the validation and results in this paper will draw attention to the behavior of techniques as a useful reference to future neuroimaging studies where appropriate.

## Materials and methods

The data set used in this study is described, followed by a description of the MR image pre-processing that we applied to all our experiments. Finally, we calculate evaluation criteria used to contrast performance of MALF, MABMIS, and BRAINSCut.

### Data description

Three subsets were investigated to assess different aspects of segmentation quality resulting from MALF, MABMIS, and BRAINSCut methods: two from PREDICT-HD (Paulsen et al., 2008), and one from THP data (Magnotta et al., 2012). PREDICT-HD collected T1-weighted (and T2-weighted) MRI data at 24 sites. The sites involved in PREDICT-HD had Siemens, GE, or Phillips scanners, and some sites upgraded their scanners from 1.5 to 3.0 T during the study period. For each of the data used in this study, a summary of data is given in Table 2 and the detailed image acquisition protocol is described elsewhere (Paulsen et al., 2008; Magnotta et al., 2012). A subset of 35 scans with manual traces (PHD35), THP multicenter data (THP), and 13 subjects of a longitudinal (L-PHD13) data set were used to assess segmentation quality in terms of accuracy, multicenter reliability, and longitudinal consistency, respectively (see Table 2).

**Table 2:** **Image profile for three subsets used in this study is presented**.

	***n***	**Notes**	**Scanner type each scan**	**TE (ms, T1 w)**	**TE (ms, T2 w)**	**Field strength**
PHD35	35	1 scan/subject	GE (2)	2.804, 2.82	88.919, 79.97	3.0 T
			Siemens (28)	1.93~3.09	430,433	
			Phillips (5)	3.5	182.566~185.971	
THP	5	Repeated scans at 8 sites per subject	Siemens (5)	(Magnotta et al., 2012)		3.0 T
			Phillips (3)			
L-PHD13	13	8~10 longitudinal scans per subject	GE (49)	3, 5	28~98	1.5 T/3.0 T
			Siemens (175)	2.87~4.75	430~4800	
			Phillips (5)	2.925~3	NA	

*The detailed image acquisition protocol is described elsewhere (PHD: Paulsen et al., 2008; THP: Magnotta et al., 2012)*.

#### PHD35

The 35-scan set of PREDICT-HD was selected to assess segmentation accuracy against manual traces. Thirty-five scans were selected by varying acquisition site as well as tissue ratio, which is generally thought to correlate with brain atrophy. Their MRI scans were manually delineated for all 12 structures of interest: the accumben nucleus, caudate nucleus, globus palladium, putamen, thalamus, and hippocampus in the left and right hemispheres.

#### THP

THP data from a multicenter reliability study (Magnotta et al., 2012) was incorporated into this study to compute measurement variation from MRI-driven volumetric measurements with multicenter data collection from the same subjects. The THP data provides a series of repeated scans of fives subjects at eight different sites over a short time period where biological changes would be negligible.

#### L-PHD13

Longitudinal reliability within subjects utilizes 13 PREDICT-HD subjects that repeatedly collected MR data for more than three time points. The subjects were also selected to include various disease burden statuses [CAG-Age Project or CAP score (Zhang et al., 2011b)], which are generally known to have different brain atrophy levels.

### Image processing

MR images were pre-processed using tools from BRAINSTools suite. Preprocessing of MR images consists of AC-PC spatial alignment (Lu, 2010; Ghayoor et al., 2013), co-registration between T1-weighted (T1-w) and T2-weighted (T2-w) images, and multimodal bias-field correction (Kim and Johnson, 2013).

The segmentation was performed on the bias-field corrected T1-w and T2-w images. The automatic segmentation tools used in this study are all publicly available and their characteristics are summarized in Table 1.

### Evaluation

To evaluate the performance of the subcortical segmentation results, we analyzed their accuracy, multicenter reliability, and longitudinal reliability using three sets of *in-vivo* MRI data.

Segmentation accuracy in this study is a measure of how similar automated segmentation is compared to manual segmentation (the *de facto* gold standard). Using a 10-fold cross-validation approach, Dice Similarity Coefficient (DSC), and intraclass correlation (ICC) were computed by contrasting automated segmentation against manual traces. For 10-fold cross-validation, 35 subjects are roughly subdivided into 10 subsets (three or four subjects per set) and cross-validation is conducted to estimate accurate segmentation performance (more details available in the Supplemental Materials). DSC is a measure of how much two segmentations overlap in volume, and a higher DSC indicates a better correspondence in volume between two raters. ICC measures a correlation between two independent approaches on a series of data, and the approaches are generally accepted as equivalent if the ICC is higher than 0.75 (Shrout and Fleiss, 1979). A higher DSC and ICC together indicate better segmentation accuracy when compared to the gold standard.

To assess multicenter reliability, the coefficient of variation (CV) was calculated as: CV% = (SD volume/Mean volume) ^*^ 100. The CV was calculated from these THP scans. Note that the CV does not measure the correctness of segmentation, only the variability of segmentation algorithm across sites within subjects. CV compares the variability of a measurement to it's mean, which gives a much better idea of the signal than assessing each alone (large variability along with a large mean is not as worrisome as large variability with a small mean).

We attempted to quantify the longitudinal reliability using Akaike Information Criterion (AIC) from the restricted maximum likelihood (REML) approach assuming linear changes in subcortical volumes, if they exist, in the course of disease progression. As mentioned in DeShon et al. (1998), longitudinal data present many challenges for analyses and, in particular, the estimation of reliability. Thus, we also reviewed a visualization of the trajectory of each volume to ensure that the AIC, as a longitudinal reliability estimate, does not bias the interpretation of our results in any undesirable direction; the AIC can only be considered as a measure of the longitudinal reliability of the tool when the approach presents valid segmentation (higher DSC and ICC) and smooth trajectory on the plot.

## Results

### Segmentation accuracy

**Figure 1:**
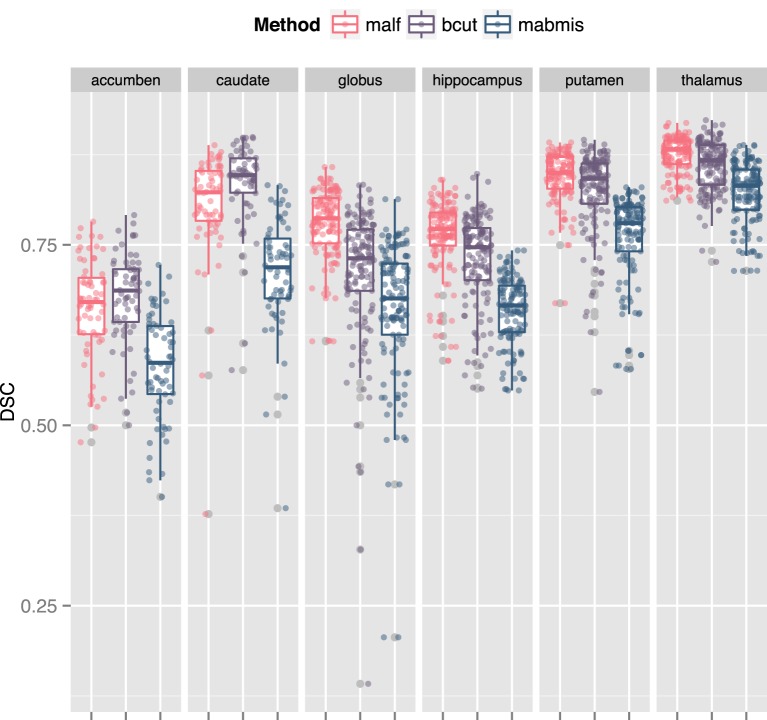
**Comparison of the Dice Similarity Coefficient (DSC) of three methods to the manual traces**. A higher DSC indicates better accuracy. For all six subcortical stuctures, BRAINSCut, and MALF presented higher DSC values than MABMIS. Other than accumbens and caudates, MALF had higher DSC values than BRAINSCut.

**Figure 2:**
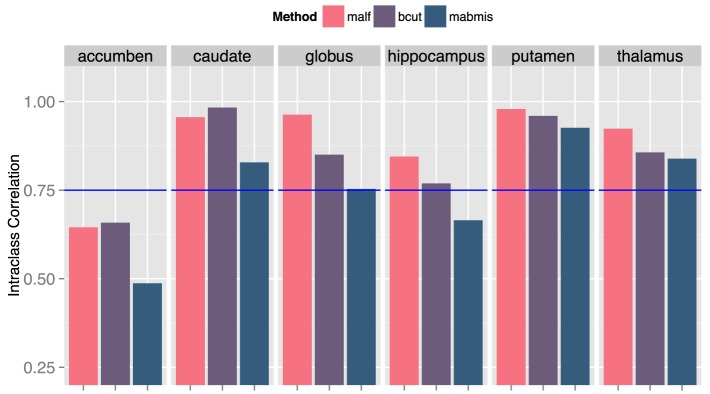
**Comparison of the intraclass correlation (ICC) of three methods to the manual traces**. A higher ICC means better correspondence with the manaul traces and, therefore, improved accuracy. ICC = 0.75 (blue line) was a suggested bound by Shrout et al. (Shrout and Fleiss, 1979) for two independent measruements to be equivalent.

The three methods explored differed for segmentation accuracy as measured by DSC (Figure 1) and ICC (Figure 2) against manual segmentation. For all subcortical structures, BRAINSCut and MALF presented higher DSC and ICC than MABMIS. Furthermore, MALF had generally higher DSC and ICC than BRAINSCut.

### Reliability across centers

**Figure 3:**
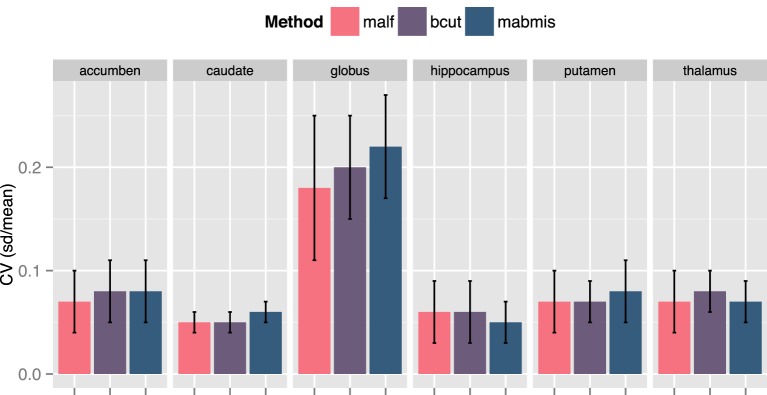
**A comparison of the Coefficient of Variation (CV) for the segmentation approaches from traveling human phantom (THP) data**. MALF presented the lowest CV, the better reliability across centers, in general. BRAINSCut (bcut) outperformed only for hippocampus in terms of multicenter reliability measured in CV.

The multicenter reliability investigation is summarized in Figure 3. Segmentations from BRANSCut and MALF had lower CV values than those obtained from MABMIS in all subcortical structures (Figure 3). This clearly indicated higher reliability for BRAINSCut and MALF. BRAINSCut presented lower CV values for hippocampus segmentation, while for all other regions, MALF presented lower CV values than BRAINSCut. An examination of CV revealed a significant improvement of multicenter reliability when using MALF rather than BRAINSCut or MABMIS.

### Longitudinal reliability within subjects across disease burden

**Figure 4:**
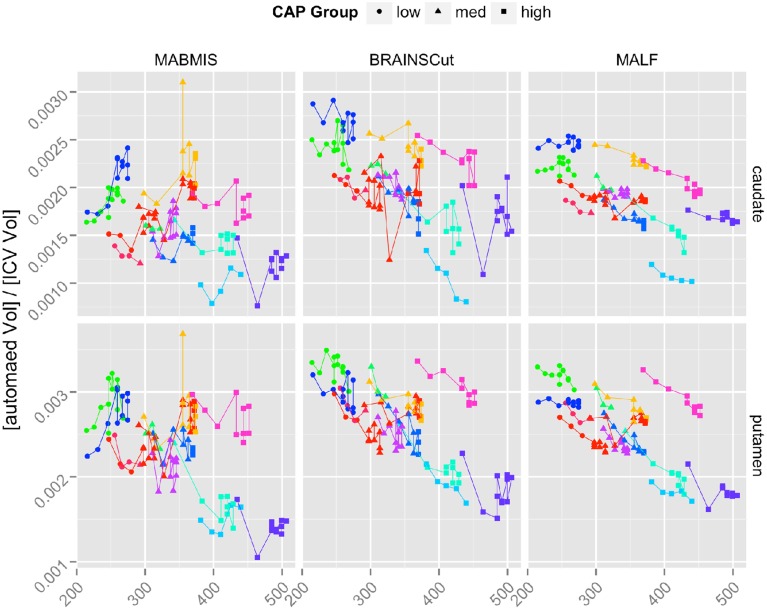
**The longitudinal trajectories of caudate and putamen volumes in left hemisphere according to Low (Circle), Medium (Triangle), and High (Square) CAP groups**. Each line segment represents one of 13 longitudinal subjects. The results of all six subcortical structures are also provided in the Supplemental Figure.

Longitudinal segmentation reliability is contrasted and summarized in Figure 4 and Table 3. MALF showed a significantly smoother trajectory (Figure 4) and the smallest AIC (Table 3) (The smaller AIC, the better fit of the model) compared to BRAINSCut and MABMIS in Pre-HD subjects. Except for the accumben nucleus and hippocampus, MALF presented very stable trajectories, as shown in Figure 4 and a Supplemental Figure.

**Table 3:** **The table presents comparisons of the Akaike Information Criterion (AIC) and log-likelihoods for the longitudinal model using three different methods**.

**ROI (hemisphere)**		**AIC**			**Log Likelihood**		
		**BRAINSCut**	**MALF**	**MABMIS**	**BRAINSCut**	**MALF**	**MABMIS**
Accumben	(L)	2320.20	^*^2204.82	2542.02	−1151.10	−1093.41	−1262.01
	(R)	^*^2124.47	2180.40	2535.45	−1053.24	−1081.20	−1258.73
Caudate	(L)	3297.80	^*^2863.82	3336.75	−1639.90	−1422.91	−1659.38
	(R)	3252.29	^*^2867.40	3251.63	−1617.15	−1424.70	−1616.81
Globus	(L)	2958.17	^*^2634.54	3134.22	−1470.08	−1308.27	−1558.11
	(R)	2806.80	^*^2614.14	2992.29	−1394.40	−1298.07	−1487.14
Hippocampus	(L)	2837.26	2798.11	^*^2756.88	−1409.63	−1390.05	−1369.44
	(R)	2769.51	^*^2646.68	2786.49	−1375.75	−1314.34	−1384.25
Putamen	(L)	3195.97	^*^2982.74	3453.81	−1588.99	−1482.37	−1717.90
	(R)	3123.95	^*^2945.32	3451.46	−1552.97	−1463.66	−1716.73
Thalamus	(L)	3152.77	^*^3100.40	3383.60	−1567.38	−1541.20	−1682.80
	(R)	3122.50	^*^3055.30	3481.18	−1552.25	−1518.65	−1731.59

The minimum AICattained by the preferred model, was achieved for the structure by the approach marked with^*^.

For the caudate and putamen, which are of foremost importance in HD study (Paulsen et al., 2014a), **a sample size analysis** was conducted in order to determine the minimum sample size necessary for detecting (with 80% power) slopes equal in magnitude to those observed in the pilot data, L-PHd13, at the 0.05 significance level. The analysis was conducted only for MALF and BRAINSCut, because estimated annual change from MABMIS was positive in the pilot data. This highly suspicious result implied that volumes of putamen and caudate increased over time, which contradicted previous findings and underlying scientific consensus regarding HD. In addition, the low segmentation accuracy (Result Segmentation Accuracy) and multicenter reliability (Result Reliability across Centers) of MABMIS indicate that its segmentation outcomes are less valid for further analysis, when compared to MALF and BRAINSCut.

For each region/method of interest, the simulation proceeded as follows: intercepts and slopes (change over time) were estimated for each region/method using linear mixed models (LMMs). LMMs were used because subjects had multiple measurements, leading to correlated data within subjects. The estimated intercepts and slopes were then used to generate a data set with a fixed sample size while assuming the data follow a linear mixed model (Psioda, 2012). This means that we used the pilot study estimates as population parameters, and is analogous to sample size analyses utilized in simpler settings, i.e., assessing differences in means at one observation time, where pilot data are used to infer a value for the variance (σ^2^). Next, an approximate *Z*-test was employed in order to test the null hypothesis **H_O_**: slope equals zero. This process was repeated M times, and the percentage of cases for which the null hypothesis was rejected was calculated, i.e., *R/M*, where *R* was the number of simulated samples for which the null was rejected. The number M used for all analyses was 1000, and this process was repeated for a sequence of sample sizes that increased in magnitude, i.e., 10, 20, 30, etc. In order to adjust for missing data, the minimum sample size with adequate power was inflated, assuming 10% missing data as shown in Tables 4, 5. In specific, the final sample size necessary was $N_{1}=\frac{1}{0.9}N_{0}$, where *N*_0_ was the minimum uninflated sample size with adequate power, and *N*_1_ was the sample size adjusted for missing data.

**Table 4:** **Estimated intercept and slope for the power analysis and sample size estimation for caudate and putamen in left and right hemisphere**.

**Method**	**Side**	**Structure**	**Intercept *[V_p_* = *V_ROI_*/*ICV (%)]***	**Slope [*V_p_*/*Year*]**
MALF	Left	Putamen	0.2748	−0.0026
		Caudate	0.1989	−0.0014
	Right	Putamen	0.2672	−0.0023
		Caudate	0.1992	−0.0014
BRAINSCut	Left	Putamen	0.2894	−0.0038
		Caudate	0.2123	−0.0029
	Right	Putamen	0.2767	−0.0031
		Caudate	0.2204	−0.0041

*The power and sample size analysis performed on data expressed as a percent of total intercranial volume along years offollow-up. Note that slope magnitude from BRAINSCut is larger than that estimated by MALF. These slopes and intercepts were utilized as population parameters in the sample size estimation with 80% power. (V_p_, Percent volume of total ICV; V_ROI_, Volume of region of interest; ICV, intercranial volume)*.

**Table 5:** **Sample size estimation to attain 80% power for caudate and putamen is reported**.

**Required sample size *N***	**Putamen**		**Caudate**	
	**Left**	**Right**	**Left**	**Right**
MALF	45	56	62	84
BRAINSCut	45	62	123	62

*The slope and intercept from Table 4 were used. Numbers are inflated from the minimum required sample size found in thepower analysis that is reported in the supplement*.

The left and right hemispheres were analyzed separately, under both the MALF and BRAINSCut segmentation methods. The following table summarizes the necessary sample size for detecting differences observed in the pilot data with at least 80% power.

## Discussion

We sought to characterize the performance of three different automated segmentation tools using *in-vivo* MRI data focusing on the potential of multi-atlas labeling approaches for use in large-scale multicenter longitudinal studies. Segmentation accuracy, multicenter reliability, and longitudinal reliability were investigated. Several major findings emerged: (1) multi-atlas labeling methods can improve the segmentation outcome in general when considering segmentation accuracy, multicenter reliability, and longitudinal reliability; (2) among the three methods, MALF performed the best for most structures, followed by BRAINSCut and then MABMIS; and (3) the sample size analysis for detection of a decrease in volume of caudate and putamen serves as a useful guide for future researchers who want to assure adequate power for detecting structural volume changes. It is worth noting that MALF did not outperform the other methods for all the structures, while our results show widespread improvement of segmentation quality using MALF. We suggest that the multi-atlas labeling approach can be one of the main focuses of future studies.

We found an increase in the DSC and ICC and a decrease in the CV for most structures when using MALF, indicating that better segmentation quality can be obtained using MALF as compared to BRAINSCut or MABMIS. A higher DSC indicates greater segmentation similarity between the automated and manual methods (the gold standard) that is usually interpreted as providing better accuracy. Furthermore, ICC, which is a measure of how two independent measures resemble each other, was increased for most subcortical structures of interest by using MALF. This increase in ICC, which has been known to be sensitive to intra-method variances as well as inter-method correlation, also reflected improved measurement accuracy. CV in this study is a measure of how reliable the tools are across centers. Reduction in CV indicates better inter-center reliability, i.e., less variation between multiple measurements on the same subject acquired at five different sites. In summary, the best performance in terms of higher DSC and ICC and lower CV was achieved using MALF; BRAINSCut appeared to be better in a few cases: higher DSC and ICC for the caudate nucleus and lower CV for hippocampus. Thus, our data (higher DSC, ICC, and lower CV with MALF) suggests possible segmentation superiority of the multi-atlas labeling approach in segmenting subcortical structures from human brain MRIs.

Our result suggests that MALF benefits the segmentation outcomes the most, but the choice of methods should depend on the researchers' aims since there exists a performance variation. For example, if one considers caudate for a region of interests, the choice of methods could depend on the study design. If the study design does not involve longitudinal data collection, BRAINSCut would be a better choice because of higher segmentation accuracy, which will give more sensitive outcomes. If the study design, however, expects longitudinal data acquisition, it might be wise to use MALF as it has smoother volumetric trajectory across years (Figure 4) with accurate segmentation results based on DSC and ICC (>0.75). For accumben nucleus, however, it is yet premature to use the data as a volumetric measurements since the accuracy of segmentation is very low, regardless the choice of the method (DSC < 0.75 and ICC < 0.7). Please note accumben is a notoriously small structure to be identified from our 1.5 or 3 Tesla MRI, ~480 mm^3^, about a size of a bean, connecting caudate and putamen. A somewhat similar argument is valid for hippocampus. Although hippocampus showed better multicenter reliability with MABMIS (lower CV), hippocampus from MABMIS may not be valid enough to be used as measurements (low DSC and low ICC). In summary, we recommend prioritizing performance criteria to choose a proper method that best suites ones study design. There is no one method that is always best. That is, depending on the purpose of the study, researchers may choose the method gives better accuracy, multicenter or longitudinal reliability for a given indication of valid segmentation.

The advantages of multicenter collaboration in observational studies include increased generalizability of results, a larger sample size, and improved efficiency, as discussed in Sprague et al. (2009) and widely practiced for rare or hard-to-recruit cases, such as HD (Paulsen et al., 2008; Tabrizi et al., 2009), Parkinson's Disease (Spencer et al., 2005), and Alzheimer's Disease (Jack et al., 2008). In such multicenter studies, across-scanner variations might interfere with the detection of disease-specific structural abnormalities (Bendfeldt et al., 2012), thereby potentially limiting the use of group analysis collected at several centers. Our data shows an increase in multicenter reliability for most subcortical structures when using MALF.

Multicenter reliability is also related to the generalizability of the tool, i.e., how much data the tool can handle, since utilization of multiple centers almost always decreases the amount of homogeneity in the data, sometimes considerably. The “*boosting”* theory in machine learning can be used to explain our results regarding superior multicenter reliability when using MALF. According to this theory, a collection of weak learning algorithms, which independently perform only slightly better than random guesses, can be converted into a highly accurate and generalizable algorithm [a better bounded generalization error (Mannor and Meir, 2001)]. Thus, our data on multicenter reliability seems to be in line with the formation of strong learners based on several weak learners; that is, the multi-atlas labeling method, where an atlas can be analogous to a weak learner, can outperform other methods. This finding is also in agreement with our previous success in subcortical segmentation using the Random-forest method, which is also a boosting method (Kim et al., 2014).

Longitudinal reliability of multi-atlas labeling tools has only been investigated in a few imaging studies (Bernal-Rusiel et al., 2012; Reuter et al., 2012) and is also limited to short-term (<2–3 years) data. The superiority of multi-atlas labeling tools for cross-sectional brain morphology investigation has been confirmed in a few studies (Wang et al., 2012; Chakravarty et al., 2013; Wu et al., 2014). The previous studies in the literature on MRI segmentation quality using the multi-atlas labeling method generally describe accuracy improvement in terms of similarity to the manually traced gold standard. We found that a multi-atlas labeling approach can also improve the longitudinal reliability of subcortical segmentation.

In the present study, visual inspection showed that MALF presented a more stable trajectory of subcortical volumes than other methods in the data collected over 10 years (Figure 4). We also investigated longitudinal reliability assuming linear changes of subcortical volume in the course of disease progression in Pre-HD subjects. MALF also presented the minimum AIC, which is considered the best model fit, for all subcortical structures except for the accumben in the right hemisphere and the hippocampus in the left hemisphere (Table 3). Although our analysis of longitudinal performance on subcortical structures showed the excellence of MALF when assuming a linear trajectory, according to the longitudinal modeling suggestions in the literature (DeShon et al., 1998), careful attention should be paid as longitudinal data present many challenges for analysis. However, our trajectory plot in Figure 4 (and Supplemental Figure) demonstrates the superior stability of MALF in comparison to BRAINCut and MABMIS.

It is interesting to note that estimation accuracy and sample size may seem counter-intuitive at first: MALF requires larger sample to obtain 80% power at 0.05 significant level for caudate in right hemisphere (Table 5) even though MALF outperformed BRAINSCut in with respect to longitudinal reliability. This is because the power analysis and sample size estimation is based on segmentation results from pilot (LPH-13) data set. For both caudate and putamen in each hemisphere, the estimated slopes from BRAINSCut were larger than those estimated by MALF (Table 4). This means that the MALF sample size analysis was powered for detecting a much finer change than that for the BRAINSCut and thus MALF requires slightly larger sample size to achieve 80% power.

Before drawing conclusions, some limitations of the present study must be acknowledged. First, more implementations of the multi-atlas labeling method have to be incorporated to investigate which is the best method for subcortical segmentation. Second, the registration technique used in each segmentation tool can be investigated for better performance. Third, studies using other psychiatric conditions are required to generalize our findings beyond HD. This will allow for a better understanding of multi-atlas labeling approach behavior for the automated processing of human MRIs.

In conclusion, we have presented evidence that multi-atlas labeling methods, which fall under an emerging segmentation approach in the field, can improve segmentation quality in terms of accuracy and reliability. Other methods can also be useful, depending on the regions of interest, study design, and implementation. However, it is likely multi-atlas labeling helps to improve the overall quality of segmentation outcomes.

### Conflict of interest statement

The authors declare that the research was conducted in the absence of any commercial or financial relationships that could be construed as a potential conflict of interest.
