# Supplementary material for: Preliminary analysis using multi-atlas labeling algorithms for tracing longitudinal change
Source: Front Neurosci. 2015 Jul 14;9:242. doi: 10.3389/fnins.2015.00242 (PMC4500912; doi:10.3389/fnins.2015.00242)
Supplement: Supplementary file 1 [file Image1.PDF]

Preliminary Analysis Using Multi-atlas Labeling Algorithms for Tracing Longitudinal Change

Regina EY Kim[1], Spencer Lourens[3], Jeffrey D Long[1,3], Jane S Paulsen[4], Hans J. Johnson[2,1]

[1] Department of Psychiatry, University of Iowa, Iowa City, IA, USA
[2] Department of Electrical Engineering, University of Iowa, Iowa City, IA, USA
[3] Department of Biostatistics, College of Public Health, University of Iowa, Iowa City, IA, USA
[4] Department of Neurology, Carver College of Medicine, University of Iowa, Iowa City, IA, USA

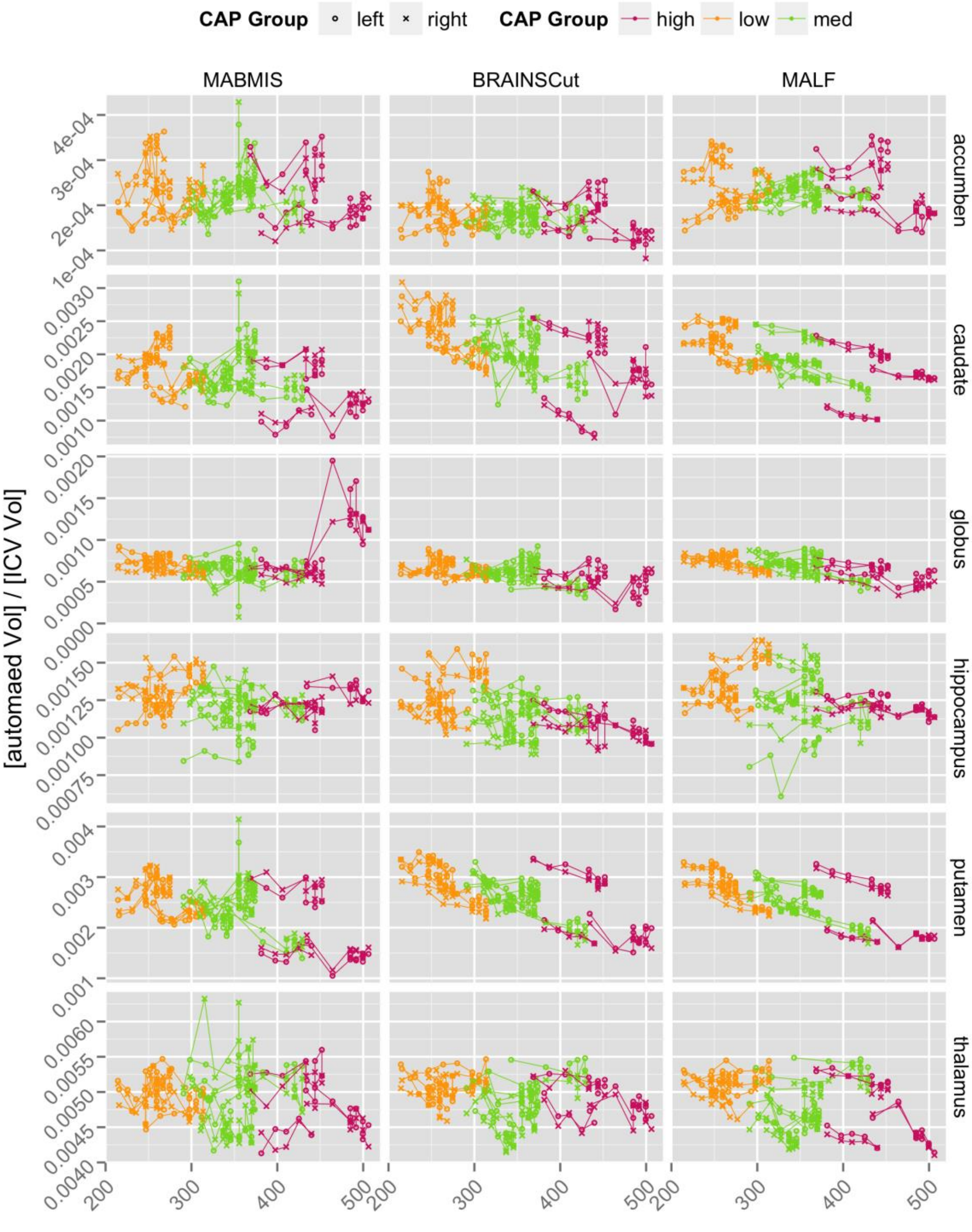

Sup. Fig. 1

The longitudinal trajectories of all six subcortical volumes according to Low (Orange), Medium (Green), and High (Red) CAP groups. Each line segment represents one of 13 longitudinal subjects.

Table 1

CAP group	Male	Female	Total
High	1	2	3
Medium	4	2	6
Low	2	2	4
Total	7	6	13

Longitudinal sample demographic (L-PHD13 subset according to the CAP group) in this study divided by CAG-Age Product (CAP) group (Zhang et al., 2011).

Table 2

Year	Baseline	1st	2nd	3rd	4th	5th	6th	7th	8th	9th	10th	11th
n	13	0	9	3	13	2	13	22	22	22	15	4

Longitudinal sample frequency by years from baseline scan (L-PHD13 subset). PREDICT-HD study plan to collect data bianually. Note that there are missing data and also repeated measure for 7th, 8th, 9th, and 10th years.

Table 3

Subject	Structure	Side	MALF		BCUT		MABMIS	
			Mean	SD	Mean	SD	Mean	SD
THP0001	Accumben	(L)	357.0	15.83	240.8	21.96	242.4	14.01
		(R)	354.6	13.13	297.8	12.93	254.2	13.29
	Caudate	(L)	3255.6	120.37	3288.0	121.18	2075.6	164.68
		(R)	3326.2	213.14	3430.2	210.90	2368.2	96.05
	Globus	(L)	1257.4	32.38	1325.0	137.66	1088.8	102.00
		(R)	1204.6	39.42	1236.8	62.17	1125.0	51.33
	Hippocampus	(L)	2110.8	95.20	1853.0	47.92	1671.2	178.36
		(R)	1970.6	86.67	1800.4	55.41	1813.8	93.72
	Putamen	(L)	4484.6	88.15	4533.4	90.77	3142.8	109.59
		(R)	4359.6	90.32	4421.6	161.05	3330.0	141.68
	Thalamus	(L)	7485.6	189.23	7397.0	158.08	6509.4	275.28
		(R)	7384.8	119.04	7226.4	329.50	6170.2	176.18
THP0002	Accumben	(L)	383.8	5.36	304.0	52.20	283.0	17.03
		(R)	381.6	32.21	309.8	47.42	277.2	24.57
	Caudate	(L)	3216.4	66.52	3009.8	193.55	2449.0	118.91
		(R)	3081.0	115.82	3065.0	162.70	2603.8	96.26
	Globus	(L)	1227.0	43.93	1321.8	87.73	916.6	176.97
		(R)	1109.6	11.59	1169.0	57.46	882.4	125.32
	Hippocampus	(L)	1967.8	81.36	1805.2	80.36	1667.2	98.04
		(R)	1883.8	76.00	1727.2	65.63	1734.4	176.47
	Putamen	(L)	4542.0	70.64	4730.6	74.56	3519.4	240.99
		(R)	4353.6	79.10	4473.2	239.30	3807.6	222.73
	Thalamus	(L)	7454.2	140.54	7502.0	391.69	6585.0	676.46
		(R)	7249.2	143.94	7345.2	409.76	6591.6	775.49
THP0003	Accumben	(L)	411.4	23.56	346.2	23.72	266.6	22.74
		(R)	411.4	19.63	343.0	18.88	300.0	15.70
	Caudate	(L)	4112.4	83.82	3890.0	151.89	2755.4	165.72
		(R)	4127.2	28.68	3937.0	178.43	2999.4	165.54
	Globus	(L)	1293.2	38.48	1303.8	129.26	978.0	246.91
		(R)	1207.8	13.37	1285.4	79.08	1032.4	178.52
	Hippocampus	(L)	1607.2	35.04	1417.2	66.80	1475.8	57.29
		(R)	1417.2	46.03	1388.8	46.11	1368.4	30.54
	Putamen	(L)	4381.4	44.69	4642.2	105.83	2904.4	208.73
		(R)	4192.8	56.80	4199.2	69.03	3332.4	280.87
	Thalamus	(L)	7836.4	59.76	7731.0	247.66	6007.4	479.83
		(R)	7540.6	69.94	7561.8	109.78	5835.6	395.55
THP0004	Accumben	(L)	421.4	23.86	340.2	21.97	325.0	43.42
		(R)	381.8	18.59	320.4	23.34	302.4	16.13

	Caudate	(L)	3705.2	35.54	3658.8	140.55	2825.8	218.50
		(R)	3816.2	92.70	3603.4	298.65	2938.2	168.40
	Globus	(L)	1475.4	24.76	1492.4	107.67	1038.6	254.95
		(R)	1403.6	25.17	1485.8	90.96	1063.6	153.98
	Hippocampus	(L)	1826.0	87.59	1739.2	61.01	1688.4	82.59
		(R)	1729.8	81.94	1731.0	38.65	1614.2	41.47
	Putamen	(L)	4895.2	36.31	4959.4	79.01	3713.8	110.60
		(R)	4583.6	42.83	4526.4	130.67	3771.2	384.08
	Thalamus	(L)	7988.8	93.85	7854.0	376.16	6987.8	376.05
		(R)	7725.0	62.97	7817.6	359.02	6555.6	391.13
THP0005	Accumben	(L)	357.2	18.21	294.2	36.73	256.0	19.35
		(R)	385.2	16.98	316.2	14.02	265.2	15.64
	Caudate	(L)	2837.0	27.10	2596.8	69.48	2024.4	95.90
		(R)	2734.8	48.26	2684.6	129.95	1996.2	80.42
	Globus	(L)	1290.2	34.02	1311.2	90.41	912.2	237.15
		(R)	1173.6	34.98	1320.8	79.87	898.6	193.47
	Hippocampus	(L)	2049.0	49.48	1870.4	31.86	1682.4	144.90
		(R)	1945.4	66.05	1978.0	17.99	1666.8	100.44
	Putamen	(L)	5126.8	34.34	5233.2	80.69	3538.4	332.81
		(R)	4960.2	41.41	4904.2	122.89	3646.4	319.06
	Thalamus	(L)	7147.8	137.08	6895.8	205.87	6336.0	684.84
		(R)	7021.8	134.71	6902.0	238.80	5904.8	354.34

Multicenter reliability results in terms of volume mean and standard deviation are presented for traveling human phantom (THP). A comparison of the mean and standard deviations for six subcortical volumetric measurements using three different automated segmentation approaches is summarized in FIGURE 3

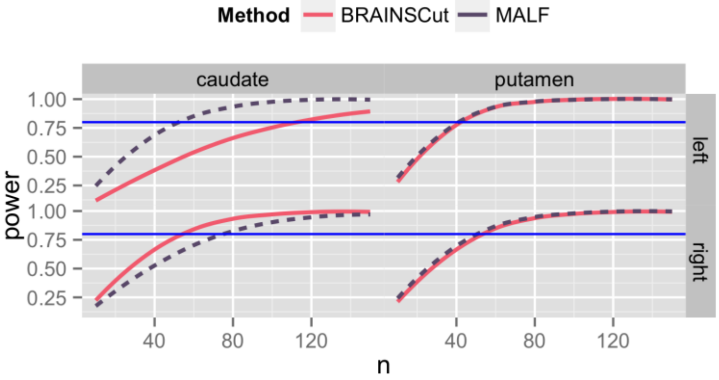

Figure 1

Power graphs using BRAINSCut and MALF for putamen and caudate in left and right hemispheres based on the estimated slope and intercept from simulation. The minimum sample size necessary for each of the above methods/regions is inflated assuming 10% missing data.

Table 4

Cross Validation Subset List. The data is publicly available at http://slicer.kitware.com/midas3/folder/2806

Set	Test	Training
Set0	7030 7017 7011	7024 7026 7028 7033 7008 7007 7002 7022 7025 7013 7014 7010 7035 7032 7027 7021 7005 7034 7020 7018 7016 7006 7023 7012 7001 7004 7009 7031 7003 7015 7019 7029
Set1	7024 7026 7028	7033 7008 7007 7002 7022 7025 7013 7014 7010 7035 7032 7027 7021 7005 7034 7020 7018 7016 7006 7023 7012 7001 7004 7009 7031 7003 7015 7019 7029 7030 7017 7011
Set2	7033 7008 7007	7002 7022 7025 7013 7014 7010 7035 7032 7027 7021 7005 7034 7020 7018 7016 7006 7023 7012 7001 7004 7009 7031 7003 7015 7019 7029 7030 7017 7011 7024 7026 7028
Set3	7002 7022 7025	7013 7014 7010 7035 7032 7027 7021 7005 7034 7020 7018 7016 7006 7023 7012 7001 7004 7009 7031 7003 7015 7019 7029 7030 7017 7011 7024 7026 7028 7033 7008 7007
Set4	7013 7014 7010	7035 7032 7027 7021 7005 7034 7020 7018 7016 7006 7023 7012 7001 7004 7009 7031 7003 7015 7019 7029 7030 7017 7011 7024 7026 7028 7033 7008 7007 7002 7022 7025
Set5	7035 7032 7027 7021	7005 7034 7020 7018 7016 7006 7023 7012 7001 7004 7009 7031 7003 7015 7019 7029 7030 7017 7011 7024 7026 7028 7033 7008 7007 7002 7022 7025 7013 7014 7010
Set6	7005 7034 7020 7018	7016 7006 7023 7012 7001 7004 7009 7031 7003 7015 7019 7029 7030 7017 7011 7024 7026 7028 7033 7008 7007 7002 7022 7025 7013 7014 7010 7035 7032 7027 7021
Set7	7016 7006 7023 7012	7001 7004 7009 7031 7003 7015 7019 7029 7030 7017 7011 7024 7026 7028 7033 7008 7007 7002 7022 7025 7013 7014 7010 7035 7032 7027 7021 7005 7034 7020 7018
Set8	7001 7004 7009 7031	7003 7015 7019 7029 7030 7017 7011 7024 7026 7028 7033 7008 7007 7002 7022 7025 7013 7014 7010 7035 7032 7027 7021 7005 7034 7020 7018 7016 7006 7023 7012
Set9	7003 7015 7019 7029	7030 7017 7011 7024 7026 7028 7033 7008 7007 7002 7022 7025 7013 7014 7010 7035 7032 7027 7021 7005 7034 7020 7018 7016 7006 7023 7012 7001 7004 7009 7031
